# Genotype and Phenotype Analyses in Pediatric Patients with *HNF1B* Mutations

**DOI:** 10.3390/jcm9072320

**Published:** 2020-07-21

**Authors:** Seon Hee Lim, Ji Hyun Kim, Kyoung Hee Han, Yo Han Ahn, Hee Gyung Kang, Il-Soo Ha, Hae Il Cheong

**Affiliations:** 1 Department of Pediatrics, Seoul National University Children’s Hospital, Seoul 03080, Korea; kkrsh@naver.com (S.H.L.); kanghg@snu.ac.kr (H.G.K.); ilsooha@snu.ac.kr (I.-S.H.); 2 Department of Pediatrics, Seoul National University Bundang Hospital, Seongnam 13620, Korea; pedkimji@gmail.com; 3 Department of Pediatrics, Jeju National University School of Medicine, Jeju 63243, Korea; hansyang78@gmail.com

**Keywords:** hepatocyte nuclear factor 1-β, *HNF1B*, congenital anomalies of the kidney and urinary tract, renal cysts and diabetes syndrome, chronic kidney disease

## Abstract

*HNF1B* mutations, one of the most common causes of congenital anomalies of the kidney and urinary tract, manifest as various renal and extrarenal phenotypes. We analyzed the genotype-phenotype correlations in 14 pediatric patients with *HNF1B* mutations. Genetic studies revealed total gene deletion in six patients (43%). All patients had bilateral renal abnormalities, primarily multiple renal cysts. Twelve patients exhibited progressive renal functional deterioration, and six of them progressed to kidney failure. The annual reduction in estimated glomerular filtration rate was−2.1 mL/min/1.73 m^2^. Diabetes developed in five patients (36%), including one patient with new-onset diabetes after transplantation. Neurological deficits were noted in three patients (21%), one with total gene deletion and two with missense mutations. Pancreatic abnormalities were more frequent in patients with missense mutations than in patients with other types of mutations. Genotype showed no significant correlation with renal outcomes or other extrarenal manifestations. The *HNF1B* scores at the times of onset and genetic diagnosis were <8 in two patients and one patient, respectively. Diagnosis of *HNF1B* mutations is clinically difficult because of extreme phenotypic variability and incomplete penetrance. Furthermore, some phenotypes develop with age. Therefore, patient age should be taken into consideration to increase the diagnostic rate, because some phenotypes develop with age.

## 1. Introduction

Hepatocyte nuclear factor-1-beta (HNF1β), also known as transcription factor-2 (TCF2), is a member of the homeodomain-containing superfamily of transcription factors [1]. During the embryonic period, early expression of HNF1β is seen in the kidneys, liver, pancreas, bile ducts, urogenital tract, lungs, thymus, and gut, where it plays an important role in the normal development of these organs [2]. Accordingly, heterozygous mutations in *HNF1B* result in a variety of phenotypes involving multiple organs [2,3,4]. 

The kidneys are the most commonly affected organs, and renal manifestations encompass a wide spectrum of congenital abnormalities of the kidney and urinary tract (CAKUT) [3,4,5]. *HNF1B* mutations are the most common monogenetic cause of CAKUT and are found in 5–38% of the patients depending on the screening policy and study design [3,5,6,7,8,9]. Cystic kidney disease, including cystic dysplasia, is the predominant form of CAKUT associated with *HNF1B* mutations [5]. Electrolyte imbalances, such as hypomagnesemia, hypokalemia, and hyperuricemia, are also a common feature of *HNF1B* mutations [3,10,11].

Almost all patients with *HNF1B* mutations have renal manifestations either isolated or in combination with extrarenal manifestations [5,6]. Maturity-onset diabetes of the young type 5 (MODY5) is one of the well-known extrarenal manifestations of *HNF1B* mutations, and the combined phenotypes of renal cysts and diabetes are referred to as RCAD syndrome [12]. Other extrarenal phenotypes include pancreatic hypoplasia with exocrine pancreatic dysfunction, genital tract malformations, abnormal liver function, and neurological disorders [3,4,5,10]. Renal and extrarenal phenotypes of *HNF1B* mutations show not only a wide spectrum but also high heterogeneity, even among individuals with the same inherited mutation [5,13,14]. No clear genotype-phenotype correlations have been demonstrated thus far. 

In this study, we analyzed the renal and extrarenal manifestations as well as genotype-phenotype correlations in pediatric patients with *HNF1B* mutations.

## 2. Methods

### 2.1. Subjects

A total of 14 unrelated Korean patients with *HNF1B* mutations who were diagnosed at the Department of Pediatrics, Seoul National University Children’s Hospital, Seoul, Korea from 2008 to 2019 were recruited in this study. These patients were identified by genetic screening of *HNF1B* gene mutations in 110 patients with CAKUT with bilateral renal involvement. The phenotypes of the patients were analyzed by a retrospective review of medical records, and the *HNF1B* score proposed by Faguer et al. Ref. [15] was calculated for each patient. Hypokalemia and hypomagnesemia were defined as serum potassium level < 3.5 mmol/L and serum magnesium level < 1.4 mEq/L, respectively [13]. Hyperuricemia was defined as serum uric acid above 7.0 mg/dL [7]. Electrolytes abnormalities were diagnosed when abnormal levels were found repeatedly or treatment was necessary. With respect to the evaluation of renal function, the estimated glomerular filtration rate (eGFR) was calculated using the Schwartz equation [16] for patients < 20 years of age and using the Chronic Kidney Disease Epidemiology Collaboration (CKD-EPI) [17] for those who were ≥20 years of age.

This study was approved by the Institutional Review Board of Seoul National University Hospital (IRB No. 0812-002-264). Informed consent was obtained from all individual participants and/or their parents.

### 2.2. Genetic Analysis

Genetic studies included Sanger sequencing, targeted exome sequencing, whole exome sequencing, and multiplex ligation-dependent probe amplification (MLPA). All coding exons with flanking introns of the *HNF1B* gene were amplified using polymerase chain reaction (PCR) followed by Sanger sequencing. PCR primer sequences are provided in Table S1. Targeted exome sequencing was designed to capture 60 genes that have been reported to cause CAKUT in humans or murine models. The detailed procedures of targeted exome sequencing, whole exome sequencing, and bioinformatic analyses have been described previously [18,19]. MLPA analysis was performed to determine the presence of copy number variations using a commercial kit (SALSA^®^ MLPA^®^ P241-E1 Probemix; MRC-Holland, Amsterdam, The Netherlands).

### 2.3. Statistical Analysis

The distribution of continuous variables is indicated by the median with range. The Mann‒Whitney test was used to compare the sizes of two independent groups, and the Kruskal‒Wallis test was used to compare the sizes of three groups. The Freeman-Halton extension of Fisher exact test was utilized to test for the difference in distribution of categorical variables between three groups. Kaplan‒Meier survival curves were employed to compare the renal survival using end-stage renal disease (ESRD) as an end point. All values are expressed as the median with interquartile range (IQR). A *p* value < 0.05 was considered to be statistically significant. IBM SPSS Statistics 20 software (IBM Co., Armonk, NY, USA) was used for all calculation.

## 3. Results

### 3.1. Baseline Characteristics

All patients were male except for Patient 1. The median ages at the times of onset and genetic diagnosis were 0.1 years (IQR 0.0–9.3) and 12.9 years (IQR 10.1–17.1), respectively. The median age at the last follow-up was 19.8 years (IQR 13.6–23.1), and the median duration of follow-up was 13.6 years (IQR 8.4–19.3). Five patients (36%) presented abnormal findings in prenatal ultrasonography, and another five patients displayed incidental azotemia with or without recurrent urinary tract infections. Analysis of family history revealed diabetes mellitus in seven patients (50%) and cystic kidney disease in three patients (21%; Table 1).

### 3.2. HNF1B Genotypes

*HNF1B* analyses revealed nine different mutations, including four missense mutations, three frame-shift short deletion mutations, one nonsense mutation, and total deletion. Five of the mutations were novel classified as “likely pathogenic” variants according to the 2015 American College of Medical Genetics and Genomics-Association for Molecular Pathology (ACMG–AMP) guidelines [20]. All mutations were unique to individual patients except for the total deletion, which was detected in six patients (43%; Table 1). 

### 3.3. Renal Phenotypes

All patients exhibited abnormal findings on renal ultrasonographic examination; multiple renal cysts were observed in 12 patients (86%), renal parenchymal hyperechogenicity was observed in nine patients (64%), unilateral multicystic dysplastic kidney was observed in four patients (29%), hydronephrosis was observed in three patients (21%), and vesicoureteral reflux was observed in two patients (14%). 

**Figure 1:**
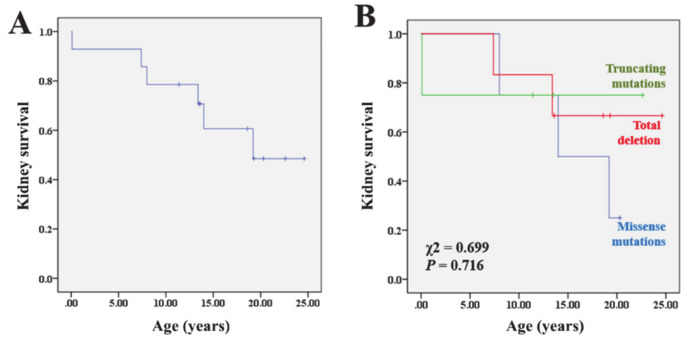
Kaplan-Meier cumulative kidney survival rates in all patients (**A**) and according to the genotype (**B**).

At the last follow-up, 12 patients (86%) showed progressive renal functional deterioration (eGFR < 90 mL/min/1.73 m^2^), including six patients (43%) who progressed to ESRD at the median age of 10.7 years (IQR 5.6–15.3; Table 1). One of the patients (Patient 6) manifested ESRD during the neonatal period. The annual reduction in eGFR, which was calculated in 13 patients using serial eGFR values measured after 2 years of age, was −2.1 mL/min/1.73 m^2^ (IQR −4.2–−1.0 mL/min/1.73 m^2^), and the reduction rates showed no significant difference between patients who progressed with ESRD (−2.1 [IQR −4.2–−1.4] mL/min/1.73 m^2^) and those who did not (−2.3 [IQR −4.2–−1.2] mL/min/1.73 m^2^). The kidney survival rates at the 10-year and 20-year follow-up were 79% and 49%, respectively (Figure 1A). 

### 3.4. Extrarenal Phenotypes and Biochemical Data

Diabetes mellitus was detected in four patients (29%) (Table 2). The median age at diabetes onset in the four patients with pretransplant diabetes was 14.6 years (IQR: 11.6–16.5). The four patients required oral hypoglycemic agents or insulin therapy to control hyperglycemia. In addition, one patient (Patient 5) developed diabetes after renal transplantation. Four patients had pancreatic abnormalities of hypoplasia or cyst, and two of them developed diabetes during the follow-up. Abnormal liver function tests and abnormalities in the hepatobiliary tract were noted in eight (57%) and five (36%) patients, respectively. Hyperparathyroidism was identified in eight patients (57%). Hypokalemia, hypomagnesemia, and hyperuricemia were observed in seven (50%), three (21%), and eleven (79%) patients, respectively. While two patients with hypomagnesemia needed magnesium supplements for 4 and 15 months, hypokalemia resolved spontaneously in all the cases without potassium replacement. Anti-hyperuricemic agents were prescribed in 8 of 11 patients.

Neurological deficits were noted in three patients (21%); mental retardation with attention deficit hyperactivity disorder was observed in two patients (14%) and delayed language development was observed in one patient (7%; Table 2).

### 3.5. HNF1B Scores

The *HNF1B* score calculated at the time of genetic diagnosis was ≥8 in 13 patients (93%) with a median value of 18 (IQR 9.5–22). Meanwhile, the *HNF1B* score at onset was ≥8 in 12 patients (86%) with a median value of 14 (IQR 9.5–16.5). The median duration between onset and genetic diagnosis was 11.1 years (IQR 1.3–14.3). 

### 3.6. Genotype-Phenotype Correlations

The patients were divided into the following three subgroups based on the genotype: patients with total deletion (TD group; n = 6), patients with missense mutations (MM group; n = 4), and patients with truncating mutations (TM group; n = 4). Hypokalemia was not observed in the MM group, while it was detected in 83% and 50% of the patients in the TD and TM groups, respectively (*p* = 0.058) (Table 3). Pancreatic abnormalities were identified more frequently in the MM group (75%) than in the TM (25%) or TD (0%) groups (*p* = 0.049). Other phenotypes, *HNF1B* scores, and renal survival rates showed no significant differences among the three groups (Table 3 and Figure 1B).

## 4. Discussion

*HNF1B* is located on chromosome locus 17q12, and the most commonly identified *HNF1B* mutation in approximately 50% of patients involves the deletion of entire genes that occurs in the context of a chromosomal microdeletion at 17q12, involving an additional 14 genes [3,21]. In our study, six of the 14 patients (43%) had entire gene deletion. Similar to many other studies, all four missense mutations detected in our study were located in the DNA binding domain of the HNF1β protein [3,5,7,22]. *HNF1B* mutations are inherited in an autosomal dominant pattern and equally affect both sexes [23]. The extreme male preponderance in our study cannot be explained and has not been reported in other studies.

The phenotypes of *HNF1B* mutations are extremely variable with inter- and intrafamilial variability, and patients with *HNF1B* mutations typically present CAKUT, either isolated or in combination with extrarenal manifestations [5,6,13,14]. Cystic disease, including cystic dysplasia, is by far the most commonly identified renal phenotype in both pediatric and adult populations in most large-scale studies [7,8,10,24], and our study corroborated this finding (bilateral multiple renal cysts in seven patients (50%) and unilateral renal cysts with contralateral multicystic dysplastic kidney or vesicoureteral reflux in five patients (36%). 

Some patients with *HNF1B* mutations present prenatally with typical findings of bilateral hyperechogenic kidneys up on fetal ultrasonography [6]. In our study, five cases were detected based on abnormal findings in prenatal ultrasonography. Electrolyte imbalances, including hypomagnesemia, hyperuricemia, and hypokalemia, are also common in patients with *HNF1B* mutations, as shown in our study. *HNF1B* is known to regulate the transcription of *FXYD2*, which encodes the γ subunit of the basolateral Na^+^/K^+^-ATPase and is involved in the reabsorption of magnesium in the distal convoluted tubule [24]. These electrolyte imbalances developed with age and became apparent in late childhood. In addition, abnormal tubular electrolyte handling associated with *HNF1B* mutations was not restricted to magnesium and potassium, but was rather consistent with a more generalized dysfunction of the distal convoluted tubule indicative of the Gitelman syndrome [11]. It is known that patients with *HNF1B* mutations usually exhibit a slowly progressive deterioration of renal function throughout adulthood, and it is known that progression to ESRD is rare in childhood [3,4]. While a median yearly decline in the eGFR of −2.45 mL/min/1.73 m^2^ was observed in a study on adult patients [13], another study on pediatric patients [25] revealed a mean annual GFR reduction of −1.0 mL/min/1.73 m^2^. However, a subset of patients develop ESRD at ages < 2 years. Compared to those in other studies, the patients in our study showed poorer renal functional prognosis; a majority (86%) presented a CKD stage ≥ 2, and half of them had ESRD. The median age of ESRD onset was 10.7 years (IQR: 5.6–15.3) in five patients, and the remaining patient had ESRD during the neonatal period. The annual reduction in eGFR was −2.1 mL/min/1.73 m^2^ (IQR −4.2–−1.0), and the cumulative kidney survival rates at the 10-year and 20-year follow-up were 79% and 49%, respectively.

Diabetes mellitus is the most frequent extrarenal phenotype associated with *HNF1B* mutations. However, diabetes in patients with *HNF1B* mutations rarely develops in childhood but rather manifests in the third or fourth decades of life [3]. While 24% of the patients developed diabetes at a mean age of 12 years (range: 10–14) in a UK study [26] on pediatric patients, only 6% of the patients in a Japanese study [7] developed diabetes in their adolescence. In our study, similar to the UK study, four patients (29%) developed diabetes at a median age of 14.6 years (IQR 11.6–16.5). In addition, one patient developed diabetes after renal transplantation, i.e., new-onset diabetes after transplant [27]. There was no difference in renal outcomes between patients with diabetes and those without diabetes in our study. 

Diagnosis of *HNF1B* mutations is clinically difficult because of extreme inter- and intrafamilial phenotypic variability, incomplete penetrance, a 50–60% *de novo* mutation rate, lack of pathognomonic findings, and wide overlap with other conditions [15]. Therefore, Faguer et al. [15] developed a *HNF1B* score as a pivotal clinical tool to provide a more rational approach to selecting patients for *HNF1B* screening. The score can be calculated based on 17 items, including antenatal discovery, family history, and organ involvement (kidneys, pancreas, liver, and genital tract). The authors suggested a score ≥ 8 as a discriminator between mutation-positive and mutation-negative patients (sensitivity 98.2%, specificity 41.1%, and negative predictive value over 99%) [15]. In a Japanese study, four out of 33 patients (12%) with *HNF1B* mutations had a score < 8 [7]. A UK study validated the clinical utility of this score in a large number of referrals for *HNF1B* genetic testing and obtained a negative predictive value of 85% by applying a cutoff score of ≥8 [23]. In a follow-up UK study, *HNF1B* scores were recalculated after adjustment for the latest available magnesium concentration, and the percentage of patients with a score ≥ 8 increased to more than 90% in the older age group [11]. In our study, two patients (14%) at onset and one patient (7%) at the time of genetic diagnosis had a *HNF1B* score < 8 and would have been not eligible for genetic testing when a cutoff score of ≥8 was applied. These findings suggest that the application of the score in younger children may wrongly predict the absence of a mutation because of later development of electrolyte imbalances as well as a lack of renal pathological findings and imaging findings of the pancreas and genital tract. Therefore, a revised scoring system or lowering of the cutoff score may be required for younger pediatric patients.

It is known that there are no clear genotype-phenotype correlations in patients with *HNF1B* mutations, which is consistent with consideration of haploinsufficiency as the disease mechanism [3]. However, some studies have shown more favorable renal functional outcomes in patients with gene deletion compared to those in patients with intragenic mutations in *HNF1B* [7,22]. Possible explanations for these findings include a dominant negative effect of non-deletion mutations or the involvement of other genes located in the deletion interval on the chromosome locus 17q12.3 [25]. Our study showed no correlation between genotype and renal functional outcomes but higher frequencies of pancreatic abnormalities in patients with missense mutations. However, we cannot generalize these findings with small numbers of our patients with *HNF1B* mutations. One study found a higher frequency of hypomagnesemia in patients with gene deletion [7]. Another study revealed roughly similar prevalence of hypokalemia and hypomagnesemia in patients with gene deletion and other mutations [13]. Interestingly, neurodevelopmental abnormalities, such as autism and developmental delays, have been reported to be associated with *HNF1B* mutations, but these complications appear to be restricted to patients with the 17q12 deletion [3,4,7,13,25]. The deleted stretch of DNA contains 14 other genes in addition to *HNF1B*; therefore, the genetic mechanism underlying these observed neurodevelopmental phenotypes is not clear [3]. However, three patients (21%) in our study had neurological deficits, but only one had a deletion mutation. Similarly, Faguer et al. [13] reported that mild but obvious mental retardation was recognized in three patients, including two with missense mutations. In other studies, a few patients with intellectual disabilities and intragenic *HNF1B* mutations have been mentioned [25,28].

## 5. Conclusions

In this study of pediatric patients with *HNF1B* mutations, most patients displayed a slowly progressive course of kidney dysfunction. Genotype did not correlate with renal functional outcomes. However, pancreatic abnormalities were more frequent in patients with missense mutations than in patients with other types of mutations. Neurological deficits were not restricted to patients with total gene deletion. Diagnosis of *HNF1B* mutations is clinically difficult because of extreme phenotypic variability, incomplete penetrance, a high *de novo* mutation rate, and wide overlap with other conditions. Furthermore, some phenotypes develop with age. Therefore, patient age should be taken into consideration to increase the diagnostic rate, especially in younger children.

## Figures and Tables

**Table 1:** Genotypes and renal phenotypes of the patients.

Pt	Age (Year) at		Mode of Onset	Family History	Mutations (REFSEQ: NM_000458.3)	Kidney Imaging	Latest eGFR
	Onset	Dx					
1	0.0	14.1	Abnormal prenatal USG	DM	c.443C > T (p.S148L)	Hypo (B), HE (B)	113 ^b^
2	11.1	13.1	DM	No	c.503T > C (p.L168P)	Cyst (B), HE (B)	36
3	0.0	11.6	Incidental renal cysts	DM, Cyst	Total deletion	Cyst (B), HN (B)	105
4	0.0	12.0	Abnormal prenatal USG	No	Total deletion	Cyst (B)	80
5	0.1	16.7	Incidental azotemia	DM	Total deletion	Cyst (R), HE (R), MCDK (L), HN (R)	69 ^b^
6	0.0	0.2	Incidental azotemia	DM	c.541C > T (p.R181*)	Cyst (L), MCDK (B)	69 ^b^
7	7.1	8.6	Recurrent UTI	No	c.439C > G (p.Q147E) ^a^	Cyst (B), HE (B)	9
8	8.9	19.6	Incidental proteinuria	No	c.313G > A (p.E105K) ^a^	Cyst (R), VUR (L)	40 ^b^
9	0.0	2.7	Abnormal prenatal USG	DM	c.1103_1116del14 (p.H368Rfs*27) ^a^	Cyst (R), HE (R), MCDK (L)	53
10	0.0	12.6	Abnormal prenatal USG	No	Total deletion	Cyst (R), HE (R), MCDK (L), HN (R)	31
11	0.2	18.3	Azotemia with recurrent UTI	No	Total deletion	HE (B), VUR (B)	105 ^b^
12	0.1	15.6	Abnormal prenatal USG	DM	Total deletion	Cyst (B), HE (B)	124
13	20.9	21.4	Incidental azotemia	Cyst	c.454delC (p.Q152fs*9) ^a^	Cyst (B)	64
14	10.5	10.6	Incidental azotemia	DM, Cyst	c.1235delC (p.P412Qfs*5) ^a^	Cyst (B), HE (B)	44

^a^ Novel mutations. ^b^ Patients who received renal transplantation. Pt, patient; Dx, diagnosis; eGFR, estimated glomerular filtration rate (mL/min/1.73 m^2^); USG, ultrasonography; DM, diabetes mellitus; UTI, urinary tract infection; Cyst, multiple renal cysts; Hypo, renal hypoplasia; HE, renal parenchymal hyperechogenicity; HN, hydronephrosis; MCDK, multicystic dysplastic kidney; B, bilateral; L, left; R, right; VUR, vesicoureteral reflux.

**Table 2:** Extrarenal phenotypes of the patients.

Pt	Hypo-Kalemia	Hypo-Magnesemia	Hyper-Uricemia	DM/Pancreatic Abnormalities	Hyper-Parathyroidism	Hepatobiliary Abnormalities	Others	*HNF1B* Score ^a^
1	(−)	(−)	(−)	(+)/atrophy	(+)	(−)	Mental retardation, ADHD	30
2	(−)	(−)	(+)	(+)/atrophy	(−)	(−)	Polycythemia, obesity	20
3	(+)	(−)	(+)	(+)/(−)	(−)	aLFT	Obesity	22
4	(+)	(−)	(+)	NA	(−)	(−)		10
5	(+)	(−)	(+)	(+)/(−)	(+)	aLFT	Mild mental retardation, ADHD, deep vein thrombosis, neurogenic bladder	22
6	(+)	(−)	(+)	NA	(+)	(−)	Bilateral inguinal hernia, anisometropia, nystagmus, delayed language development	8
7	(−)	(+)	(+)	(−)/atrophy	(+)	aLFT, hepatic cysts	PDA, iliac wing brown tumor, post urethral dilatation	18
8	(−)	(−)	(+)	NA	(+)	aLFT	Obesity	5
9	(−)	(+)	(−)	(−)/tail cyst	(+)	aLFT		14
10	(+)	(−)	(+)	(−)/(−)	(+)	aLFT, CBD stone, choledochal cyst	Hypospadias, imperforated anus, urethral obstruction	24
11	(+)	(−)	(+)	NA	(+)	aLFT, choledochal cyst		8
12	(−)	(−)	(−)	(+)/(−)	(−)	(−)		18
13	(+)	(−)	(+)	(−)/(−)	(−)	aLFT, GB stone	Obesity, polycythemia	10
14	(−)	(+)	(+)	NA	(−)	(−)	Pectus excavatum	18

^a^ Calculated at the time of genetic diagnosis. Clinical phenotypes are presented as either present (+) or absent (−). Pt, patient; DM, diabetes mellitus; aLFT, abnormal liver function test; CBD, common bile duct; GB, gallbladder; NA, not available; ADHD, attention deficit hyperactivity disorder; PDA, patent ductus arteriosus.

**Table 3:** Comparison of the phenotypes according to the genotypes.

	*HNF1B* Genotypes			*p* Value
	Missense (n = 4)	Truncating (n = 4)	Total Deletion (n = 6)	
Age at onset, years	8.0 (1.8–10.6)	5.3 (0.0–18.3)	0.05 (0.0–0.13)	0.341
Age at diagnosis, years	13.1 (8.6–13.1)	10.6 (1.5–21.4)	14.1 (11.9–17.1)	0.443
Age at the last follow-up, years	20.3 (19.0–24.9)	12.5 (9.2–20.3)	20.0 (17.4–25.8)	0.183
No. of patients with				
Hypokalemia	0	2 (50)	5 (83)	0.058
Hypomagnesemia	1 (25)	2 (50)	0	0.209
Hyperuricemia	3 (75)	3 (75)	5 (83)	1.000
Diabetes mellitus	2 (50)	0	2 (33)	0.473
Pancreatic abnormalities	3 (75)	1 (25)	0	0.049
Hepatobiliary abnormalities	2 (50)	2 (50)	4 (67)	1.000
Neurologic abnormalities	1 (25)	1 (25)	1 (17)	1.000
Progression to ESRD	3 (75)	1 (25)	2 (33)	0.500
*HNF1B* score [15] at the time of				
Onset	16 (4.8–19)	12 (8.5–17)	13.5 (8.5–15.8)	0.695
Diagnosis	19 (8.5–27.5)	12 (8.5–17)	20 (9.5–22.5)	0.437

Values are expressed as median (interquartile range) and number (%). ESRD, end-stage renal disease; IQR, interquartile range.

## Supplementary Materials

The following are available online at https://www.mdpi.com/2077-0383/9/7/2320/s1, Table S1: Primers used for PCR amplification of the *HNF1B* gene.
