# Supplementary material for: Genotype and Phenotype Analyses in Pediatric Patients with HNF1B Mutations
Source: J Clin Med. 2020 Jul 21;9(7):2320. doi: 10.3390/jcm9072320 (PMC7408390; doi:10.3390/jcm9072320)
Supplement: Supplementary file 1 [file jcm-09-02320-s001.pdf]

Supplementary Material: Genotype and Phenotype Analyses in Pediatric Patients with *HNF1B* Mutations

Seon Hee Lim, Ji Hyun Kim, Kyoung Hee Han, Yo Han Ahn, Hee Gyung Kang, Il-Soo Ha and Hae Il Cheong

Table S1. Primers used for PCR amplification of the *HNF1B* gene.

Exon	Forward Primers (5′–3′)	Reverse Primer (5′–3′)	Size (Base Pair)
1	AACAGGT GTCTGGAGGCTGA	GGTGTTGGGAGAGAAGCAGAG	619
2	AGGGATGAGGTGTACCGTACAG	AACCACCAAGGCCAAATCTAC	553
3	TCTCCAGCTCCACATGCAGT	GGTCTGTGTACTTGCCCACCT	463
4	GGATTGGCCTTTTCTCTGCA	AACCAGATAAGATCCGTGGCA	473
5	GTGCCGAGTCATTGTTCCAG	CTATGGGGCTACAATGGTTCAT	338
6	CATGCCAAGGAATCGCTAAGT	AATGATTTGACCCAGGATGCA	481
7	TTACACTTGCCTCCCATGGA	CCGAGAAAGTTCAGACCCAGA	422
8	ACATGCTTTCGGGAGTTGGT	CACAACCTTTGCACATCCATG	390
9	TGGTTGAGTTGGGCATCATC	GATTGTCTGAGGTGCCAGCA	279
